# A randomized trial comparing structured and lifestyle goals in an internet-mediated walking program for people with type 2 diabetes

**DOI:** 10.1186/1479-5868-4-59

**Published:** 2007-11-16

**Authors:** Caroline R Richardson, Kathleen S Mehari, Laura G McIntyre, Adrienne W Janney, Laurie A Fortlage, Ananda Sen, Victor J Strecher, John D Piette

**Affiliations:** 1 Department of Family Medicine, University of Michigan Health System, Ann Arbor, MI, USA; 2 HSR&D Center for Excellence, VA Health Care Medical Center, Ann Arbor, MI, USA; 3 Center for Statistical Consultation and Research (CSCAR) and Department of Statistics, University of Michigan, Ann Arbor, MI, USA; 4 Center for Health Communications Research, Department of Health Behavior and Health Education, University of Michigan and University of Michigan Comprehensive Cancer Center, Ann Arbor, MI, USA; 5 Int Med-General Med, University of Michigan Health System and Michigan Diabetes Research and Training Center, Ann Arbor, MI, USA

## Abstract

**Background:**

The majority of individuals with type 2 diabetes do not exercise regularly. Pedometer-based walking interventions can help; however, pedometer-based interventions targeting only total daily accumulated steps might not yield the same health benefits as physical activity programs specifying a minimum duration and intensity of physical activity bouts.

**Methods:**

This pilot randomized trial compared two goal-setting strategies: 1) lifestyle goals targeting total daily accumulated step counts and 2) structured goals targeting bout steps defined as walking that lasts for 10 minutes or longer at a pace of at least 60 steps per minute. We sought to determine which goal-setting strategy was more effective at increasing bout steps. Participants were sedentary adults with type 2 diabetes. All participants: wore enhanced pedometers with embedded USB ports; uploaded detailed, time-stamped step-count data to a website called Stepping Up to Health; and received automated step-count feedback, automatically calculated goals, and tailored motivational messages throughout the six-week intervention. Only the automated goal calculations and step-count feedback differed between the two groups. The primary outcome of interest was increase in steps taken during the previously defined bouts of walking (lasting at least 10 minutes or longer at a pace of at least 60 steps per minute) between baseline and end of the intervention.

**Results:**

Thirty-five participants were randomized and 30 (86%) completed the pilot study. Both groups significantly increased bout steps, but there was no statistically significant difference between groups. Among study completers, bout steps increased by 1921 ± 2729 steps a day. Those who received lifestyle goals were more satisfied with the intervention (p = 0.006) and wore the pedometer more often (p < 0.001) than those who received structured goals.

**Conclusion:**

In this six-week intervention, Lifestyle Goals group participants achieved increases in bout steps comparable to the increases seen in the Structured Goals group, representing almost a mile a day of additional moderate intensity bout activity. Pedometer-based walking programs that emphasize total accumulated step counts are more acceptable to participants and are as effective at increasing moderate intensity bouts of physical activity as programs that use structured goals.

**Trial registration:**

NCT00151021

## Background

Physical activity can improve glucose control, [1-5] lower blood pressure,[1] improve blood lipid profiles, [6,7] decrease the risk of adverse cardiovascular events, [8] and decrease the risk of death among people with type 2 diabetes [9-11]. However, despite these potential health benefits, the majority of individuals with type 2 diabetes do not exercise regularly [12,13]. Among people with type 2 diabetes who do exercise, walking is the most popular form of physical activity [13].

Sedentary individuals with type 2 diabetes who are trying to start a walking program often use pedometers, small pager-like devices worn at the waist that count steps taken while worn. Pedometer-based walking programs have been shown to increase step counts in individuals with type 2 diabetes [14-16]. Typically, pedometer-based walking programs involve setting a daily step-count goal such as 10,000 steps per day, and participants are encouraged to accumulate steps throughout the day to reach their goals. In this lifestyle approach to physical activity, every step taken counts, and walking speed and duration of walks taken are not considered. Thus, it is possible to reach high step-count goals without performing bouts of moderate intensity physical activity.

Part of the reason pedometer-based walking programs are popular is because individuals can achieve their step-count goals in ways that suit their lifestyle. However, some experts argue that such lifestyle programs might not result in activity that is of sufficient duration and intensity to yield improvements in cardio-respiratory fitness, and thus such programs might not yield the health benefits of more intensive structured physical activity programs [17]. Most national guidelines recommend that people perform sustained bouts of exercise of at least moderate intensity activity, rather than just accumulating activity throughout the day without any consideration of intensity or duration [18-21]. Cardio-respiratory fitness, one of the most important predictors of adverse cardiac events and mortality, [22-24] can be improved only by physical activity of duration and intensity sufficient to induce a training effect. Activity that does not reach the training threshold might yield other health benefits, but such brief and light activity is unlikely to result in improved cardio-respiratory fitness.

Fortunately, newer enhanced pedometers that incorporate built-in clocks and memory are available, allowing detailed tracking of both duration and intensity of walking, along with total steps. These enhanced pedometers can be used to track progress toward more structured goals that include a minimum duration and intensity of walking bouts into a pedometer-based walking program. The use of these enhanced pedometers paired with structured goals may increase the likelihood that those people who can successfully meet these goals improve their cardio-respiratory fitness. However, these achievements may come at a cost in terms of participant satisfaction and adherence. Little is yet known about how structured pedometer goals might affect participant's walking patterns, satisfaction and adherence to a walking program. It is important to determine whether goals can improve cardio-respiratory fitness combined with participant satisfaction and adherence in order to develop programs that can lead to long-term health benefits for people with type 2 diabetes.

The purpose of this study was to compare two different goal setting strategies in a pedometer-based walking program for people with type 2 diabetes; one employing lifestyle goals (LG) for overall steps and the other employing structured goals (SG) that emphasize greater activity intensity. The primary outcome measure was steps taken during bouts of walking that last for at least 10 minutes at an intensity of at least 60 steps per minute. Additionally, we compared participant satisfaction and adherence to each program type. We hypothesized that SG would not significantly increase steps taken during bouts of activity, but, instead, SG would decrease participant satisfaction and adherence. Therefore, overall LG would be effective both at increasing steps taking during bouts of activity, and also at increasing participant satisfaction and adherence. People who are given LG (total steps) without any criteria for duration or intensity may choose to increase their walking by going for a long walk that meets minimum intensity and duration criteria even though such activity is neither mandatory, nor encouraged by the goal.

## Methods

### Study design

This six-week, pilot, randomized trial was conducted using an automated Internet-based intervention using uploading-enhanced pedometers for people with type 2 diabetes. All participants wore an uploading-enhanced pedometer throughout the study period, and all had access to a personally-tailored Stepping Up to Health web page that allowed them to view: tailored motivational messages, tips about managing diabetes, automatically calculated goals, and feedback about performance toward goals. Participants were randomized to one of two groups. The LG group received goals targeting total accumulated steps, as seen in a traditional pedometer-based walking program. The SG group received goals based only on steps taken during walking bouts of at least 10 minutes with at least 60 steps per minute. We were interested primarily in whether or not an increase of steps taken during these bouts would occur (and in which group), compared to the baseline period during which no goals were set.

### Study population

Participants were eligible for the study if they were at least 18 years of age and had type 2 diabetes. Eligible participants also reported regular e-mail use, and had access to an Internet-connected computer with a Windows 2000 or XP operating system and an available USB port. Participants self-reported less than 150 minutes per week of moderate physical activity at baseline and were interested in starting a walking program. Participants also had to be able to communicate in English, provide written consent, and obtain medical clearance to start a walking program from a primary care physician, endocrinologist, or cardiologist. Participants attended one face-to-face session with our study coordinator, or another member of the study staff, to complete the enrollment process. Individuals were excluded from the study if they had used a pedometer in the past 30 days or were pregnant.

### Recruitment

The potential participants were identified through advertisements and referrals. Advertisements included flyers at the University of Michigan Hospital, nearby clinics, and other public locations, an ad in the local newspaper and a listing on a medical research participant recruiting website. Additionally, water bottles with the study logo and contact information were distributed to potential participants and their physicians in order to enhance recruitment.

### Enrollment

All potential participants were briefly screened for eligibility over the telephone. Eligible and interested individuals were invited to attend a one-hour session to further discuss the details of the study, sign an informed consent document, and begin the enrollment process. After participants provided written informed consent, study staff assessed weight, height, and blood pressure, and participants received a medical clearance form to be completed by their physician; a password giving them access to the online baseline survey; a blinded, enhanced pedometer with an uploading USB cord; detailed instructions about uploading pedometer data to the Stepping Up to Health server; instructions for care and use of the pedometer; and a handout on walking safely specifically for people with diabetes. All participants obtained written medical clearance from a physician.

### Baseline data collection

Participants were told to go about their usual activities and not to try to increase their activity during the baseline data collection phase. Participants wore the study pedometer for seven days to assess baseline step counts. During this baseline data collection phase, a sticker was placed over the pedometer face to prevent participants from seeing their step counts, and participants did not receive any goals or feedback from the Stepping Up to Health website. During the baseline week, participants also completed a detailed online survey that included questions about demographics, health history, motivations and barriers for walking, knowledge and attitudes about diabetes, and comfort with computers.

### Randomization

At the end of the first week, participants who had successfully uploaded a week of baseline step-count data, completed the baseline survey, and obtained written medical clearance were randomized with equal probability into one of the two intervention groups: the LG group or the SG group. Participants received an email message informing them of their group assignment and were instructed to remove the sticker from their pedometer to unblind it. Participants were given access to a personal web page on the Stepping Up to Health website, which allowed them to view the tailored motivational messages and tips about managing diabetes. The personal web page also displayed automatically calculated goals, and feedback about performance toward goals, based on group assignment.

### Interventions

Participants were asked to wear the unblinded pedometers every day, from waking to sleeping during the six-week intervention period. Participants could read their step-count data from the pedometer display at any time throughout the day. Additionally, all participants received one new personally tailored motivational message each week on their web page, based on their individual responses to the baseline survey. The tailored motivational message algorithm was identical for the two groups, and it was developed based on aspects of the Health Belief Model (HBM) [25]. The messages highlighted perceived benefits of exercise while addressing perceived barriers and strategies to overcome those barriers. The motivational messages were therefore tailored so that the content specifically addressed the characteristics, motivations and barriers of the recipient.

**Figure 1:**
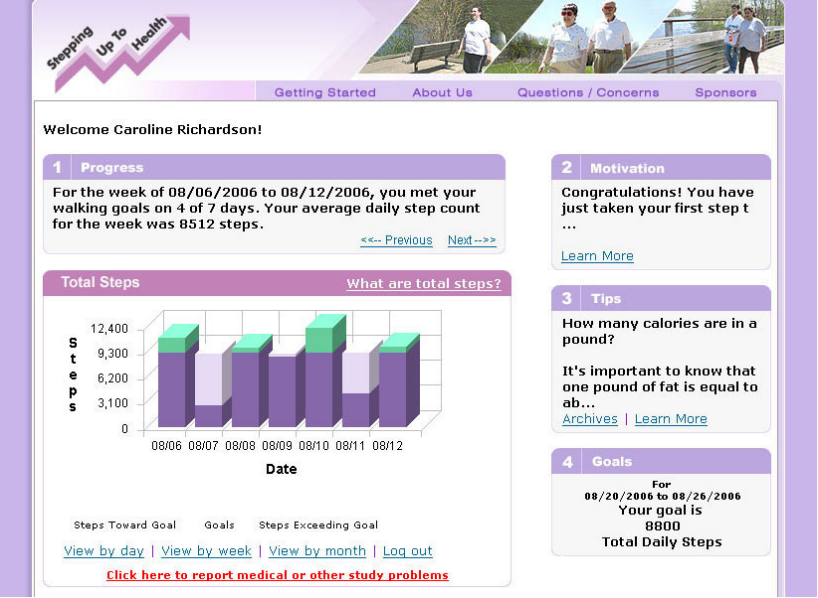
Screen shot from the Stepping Up to Health website.

Topics included in the tips about managing diabetes were diet and nutrition, controlling blood sugar, medication use, foot care, exercise, stress, and complications of diabetes. Figure 1 is a screen shot of a sample personalized web page.

### The pedometer

**Figure 2:**
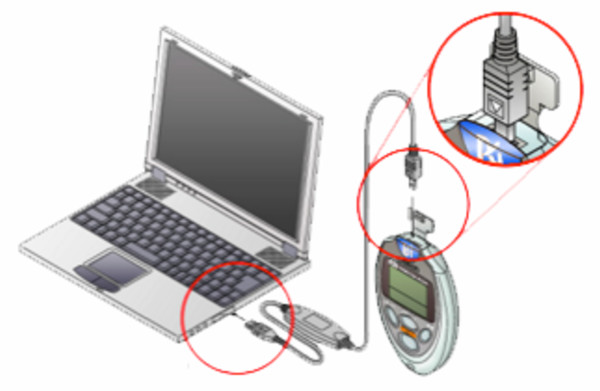
Omron enhanced pedometer system.

The pedometer worn by participants in this study was a beta test version of the Omron HJ-720IT (Figure 2). This pedometer contains a dual-axial accelerometer, an internal clock, enough memory to store 42 days of detailed time-stamped hour-by-hour step-count data, and an embedded USB port. The mechanism and algorithms used to measure total and bout steps in these pedometers have been found to be valid and reliable in previously published studies. [26-29]. For each hour of the day, the pedometer records a time and date, total steps, bout steps, and an activity flag that indicates if the pedometer detected any movement at all during the hour. While the enhanced pedometers used are accelerometer based, they do not store or upload detailed accelerometer data or data on non-walking physical activity.

Participants were instructed to connect their enhanced pedometers to the USB port of their computer at least once a week; however, as often as they desired, they could upload detailed, hour-by-hour, time-stamped step-count data to a central computer. Data were transferred automatically from the participant's computer to a central repository.

### Automated goal setting and step-count feedback

#### The LG group

Participants randomized to receive LG were instructed to focus on total accumulated steps. The algorithm for calculating LG involved averaging the previous seven days of total step data, adding 1,200 steps to the average, and then rounding off to the nearest 100 steps. LG group participants were encouraged to focus on bout steps. A computer program translated each individual's uploaded pedometer data into individualized step-count goals and feedback messages delivered through participants' personal Stepping Up to Health web page. Feedback about progress toward goals was displayed graphically and via text messages on the participants' web page. All graphs displayed total steps; success or failure in achieving goals was based only on total step counts. LG participants received credit for any and all walking during the day, even if they did not achieve minimum duration and intensity criteria. Goals were not necessarily monotonically increasing. For example, if a participant was sick, and thus had low step counts for one week, the subsequent weeks' goals might be lower than the goal for the week the participant was sick. The maximum allowable goal was 10,000 steps per day. If the calculated goals were greater than 10,000 steps per day, the goal was re-set at 10,000 steps per day.

#### The SG group

Participants randomized to receive SG were instructed to focus on bout steps. They were encouraged to set their pedometer to display bout steps (labeled aerobic steps on the Omron pedometers), and they were assigned weekly automatically calculated bout steps goals based only on bout-step data uploaded from the previous week. SG participants were encouraged to focus on bout steps. The algorithm for calculating SG involved averaging the previous seven days of bout step data, adding 800 steps to the average, and then rounding off to the nearest 100 steps. Feedback about progress toward goals displayed in graph and text format on the participants' web page. For SG participants, all graphs displayed only bout steps, and success or failure in achieving goals was based only on bout step counts. When SG participants did not meet minimum duration and intensity criteria, their graphs displayed zero steps. As in the LG calculations, SG participants were not necessarily monotonically increasing, and they never exceeded 10,000 steps.

Both the assigned goals and the goal increments were lower in the SG group than in the LG group (Tables 1 and 2). The increments for the two groups were selected based on data from our prior studies. Increments were also selected to equalize both goal difficulty and average daily total steps across the two groups. The increments for the SG group were lower because bout steps are approximately 2/3 of total steps for average participants. While those in the LG group got reinforcement and positive feedback every time they took a step, those in the SG group saw zeros on the pedometer display and in the graphical web-based feedback for days in which they did not complete a walk meeting minimum 10-minute bout criteria.

**Table 1:** Sample data uploaded from a participant's pedometer

	Sun 4/9	Mon 4/10	Tue 4/11	Wed 4/12	Thu 4/13	Fri 4/14	Sat 4/15	Total
Total Steps	3,431	475	4,328	5,524	4,930	5,957	316	21,530
Bout Steps	0	0	1,186	1,099	3,666	2,364	0	8,315

**Table 2:** A comparison of goal calculations for lifestyle and Structured Group goals.

	If randomized to the Lifestyle Goals group:	If randomized to the Structured Goals group:
Average from week of 4/9 to 4/15	3,076 total steps	1,188 bout steps
Increment	+1,200 steps	+ 800 steps
New Goal for 4/16 to 4/22 – rounded off to nearest 100's	4,000 total steps	2,000 bout steps

#### Post intervention assessment

At the end of the six-week intervention period, participants received an automated e-mail message asking them to complete the end-of-study activities, including: filling out a post-intervention online survey; scheduling the second session with the research staff to measure weight and blood pressure; and returning the pedometer. Participants who completed the study, and returned the enhanced pedometer, received a $25.00 gift certificate.

### Statistical analysis

Baseline characteristics were summarized using appropriate descriptive statistics. Means and standard deviations are reported for variables with a normal distribution and percentages are reported for categorical variables. Average daily bout steps and total steps were calculated for each participant, and comparisons were made between groups using unpaired t-tests. For pre-post comparisons, we used paired t-tests. For between-group comparisons of dichotomous and categorical data, including participant satisfaction data, we used chi square comparisons. Non-parametric statistics including the sign-rank test and the rank-sum test were used respectively with paired and unpaired data that were not normally distributed.

To examine individual patterns of walking, we estimated two linear regressions for each individual participant. One regression estimated the association between day of program and total steps, and the second regression estimated the association between day of program and bout steps. Days one to seven of the active intervention (after the baseline week) were dropped from each regression analysis because many participants achieved very high and unsustainable step counts on one or more days during the first week of using the pedometer. A participant was considered to have successfully increased their total steps if in the equation regressing total steps on the day of program, the estimated slope for the day of program was greater than 35. This translates to an increase in total steps of 35 per day of the program, i.e. an increase of approximately 1500 steps or 0.75 miles over the six-week intervention, which is deemed clinically significant. Similarly, a participant was considered to have successfully increased their bout steps if in the equation regressing bout steps on the day of program, the estimated slope for the day of program was greater than 35. All statistical analyses were carried out using STATA 9.0 (StataCorp, College Station, Texas).

### Qualitative analysis

There were several open-ended participant satisfaction questions in the final survey. The responses to these questions were analyzed and coded using standard qualitative methods. Common themes were identified and differences in themes between groups were examined.

### Human subjects and safety issues

This study was approved by the University of Michigan Institutional Review Board. All participants signed written informed consent documents.

## Results

### Recruitment and retention

**Figure 3:**
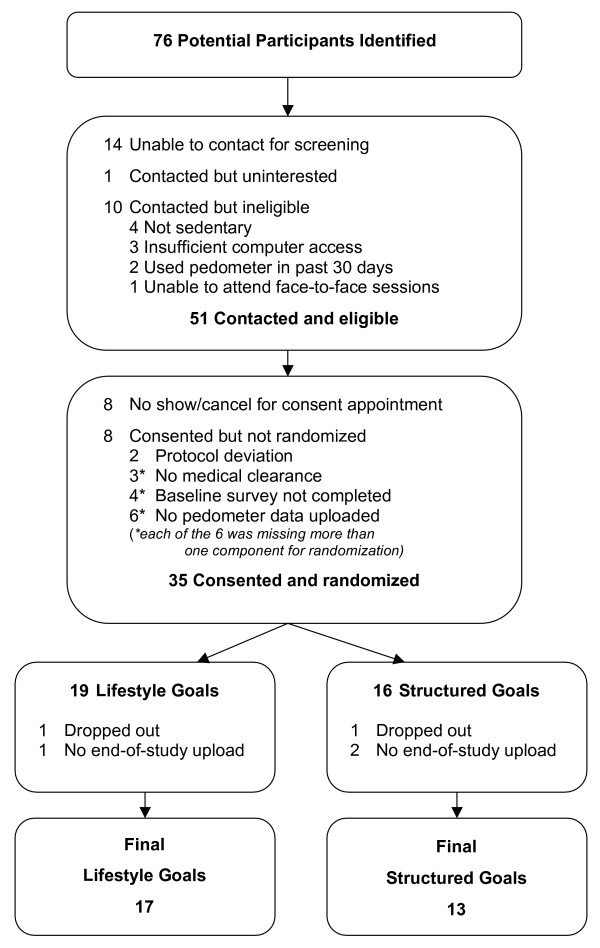
Recruitment and Retention.

A total of 76 participants were identified through community-based recruitment and physician referral. Of those, 62 were screened and 51 were eligible to participate in the study. Of those who were eligible, 35 completed baseline enrollment procedures and were randomized (LG group = 19, SG group = 16). Thirty participants (86%) completed the entire six-week intervention. Details of recruitment and retention are reported in Figure 3 and baseline characteristics of the sample are described in Table 3. Participant ages ranged from 38 to 71 years. Five participants did not complete the program. Two participants dropped out due to lack of interest in the program, one from each group. Three participants failed to upload pedometer data at the end of the study, one from the LG group and two from the SG group.

**Table 3:** Baseline and demographics by randomization group.

	**Total Step Group**	**Bout Step Group**
**N**	17	13
Age (SD)	52 ± 12	53 ± 9
Sex		
Male	29%	38%
Female	71%	62%
Race/Ethnicity		
White	76%	77%
Black	18%	8%
Other	6%	15%
Baseline Average Daily Step Count	4,157 ± 1,737	5,171 ± 1,769
Baseline BMI	38.6 ± 8.2	35.3 ± 8.6
Baseline Blood Pressure		
Systolic	133 ± 18	136 ± 12
Diastolic	80 ± 9	82 ± 11
On Insulin		
No	88%	69%
Yes	12%	31%
Income		
<$30,000	18%	8%
$30,000-<$70,000	18%	31%
≥ $70,000	65%	62%
Education		
HS diploma or GED	6%	8%
Some college	47%	15%
College degree	18%	46%
Graduate degree	29%	31%
Internet Usage (Home)		
Never	6%	23%
≤ 4 times per month	12%	8%
Several times a week	12%	23%
Almost every day	71%	46%
(Work)		
Never	29%	23%
≤ 4 times per month	6%	8%
Several times a week	0%	8%
Almost every day	65%	62%

SD = Standard Deviation

### Primary outcome – average bout step counts

Both groups significantly increased their average daily bout steps between baseline and the end of the intervention period; however, there were no between-group differences in bout step increases. Collapsing across groups, study completers increased their average daily bout steps by 1921 +/- 2729 steps (paired t test p = 0.0006). Additionally, there was no difference in increase in total steps between those who received LG and those who received SG. Study completers increased their average daily total steps by 1938 ± 3298 steps (pre-post paired t-test p = 0.0032). For participants in both groups, most of the increase in walking was achieved during bout steps. Details of the average step-count increases are presented in Table 4.

**Table 4:** Step-count results overall and by group for both total and bout steps.

	**All Participants**	**Lifestyle Goals Group**	**Structured Goals Group**	**p-value (between-group)**
**N**	30	17	13	

**Total Steps**				
Pre-Intervention	4,596 ± 1,794	4,157 ± 1,737	5,171 ± 1,769	0.1272
Post-Intervention	6,534 ± 3,456	6,279 ± 3,306	6,868 ± 3,751	0.6520
Change	1,938 ± 3,298	2,122 ± 3,179	1,697 ± 3,564	0.7329
p-value (pre/post)	0.0032*	0.0142*	0.1117	

**Bout Steps**				
Pre-Intervention	386 ± 691	286 ± 599	516 ± 801	0.3768
Post-Intervention	2,306 ± 2,734	2,070 ± 2,814	2,616 ± 2,706	0.5961
Change	1,921 ± 2,729	1,783 ± 2,741	2,101 ± 2,815	0.7583
p-value (pre/post)	0.0006*	0.0164*	0.0196*	

* = p < 0.05

Step counts expressed as Mean ± Standard Deviation

P values reflect two tailed paired t-tests

**Figure 4:**
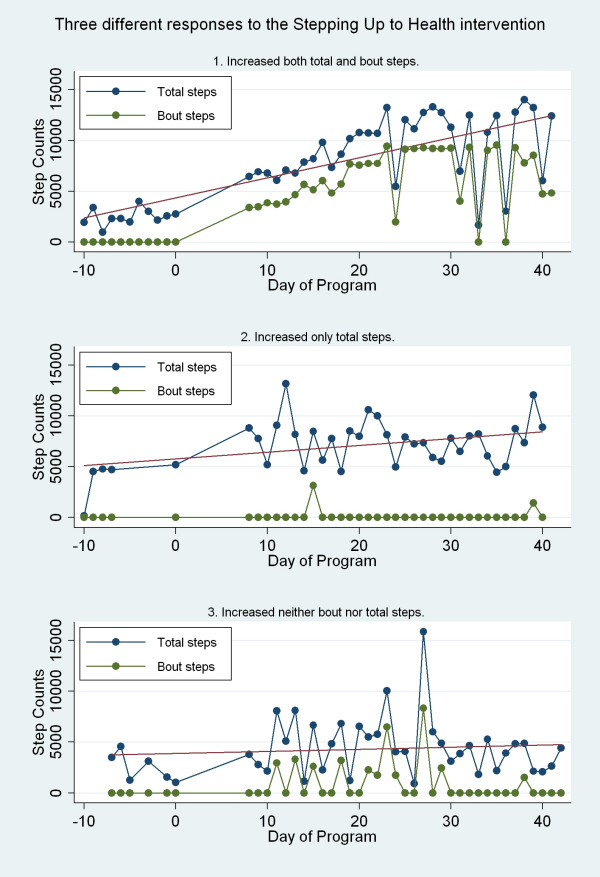
Walking patterns for three sample participants.

Analysis of walking patterns for each individual participant show that approximately 44% of participants successfully increased their average daily steps, and 37% successfully increased their average daily bout steps. Table 4 presents more details on this individual level analysis by group, and Figure 4 presents sample step-count graphs for individual participants in each of these response categories.

All SG participants who successfully increased their total steps did so by increasing bout steps. Seventy-five percent of LG participants who successfully increased their total steps did so by increasing bout steps. While only one participant from each of the two groups accumulated 150 minutes of bout activity during the baseline week, six participants in each group, or 40% of the participants, accumulated 150 minutes of bout activity during the final week of the program (for all participants pre-post chi p = 0.07).

### Participant satisfaction

LG participants reported higher satisfaction, with 100% reporting that they would definitely recommend the Stepping Up to Health program to friends, while only 62% of SG group would definitely recommend the program to a friend (chi sqr p = 0.006). In the LG group, 71% of participants found the graphs "very useful" compared to 31% in the SG group (chi sqr p = 0.03). In response to open-ended questions about satisfaction, almost all participants commented that they liked tracking their steps with the pedometers and seeing their graphs. Participants in the SG group reported being discouraged by not getting any credit for shorter walks. One SG group participant stated, "It was frustrating when I walked and walked, and it didn't count because of the 10-minute bouts."

### Adherence

For 30 participants in a 42-day trial we expected 1260 days of uploaded data during the intervention. A total of 21 or 1.7% of expected days were not uploaded due to technical or logistical issues including pedometer battery failure. Additionally, 99 days had activity for less than three hours or had less than 100 steps for the day suggesting that the pedometer was not worn at all on those days. SG participants were five times as likely to fail to wear the pedometer than the LG participants (15% versus 3%, p < 0.001). On those days that the pedometer was worn, the SG group wore the pedometer for fewer hours of the day on average than the LG group. The SG group wore the pedometer for a mean of 14.5 hours per day and the LG group wore the pedometer for a mean of 16.5 hours per day (t-test p = 0.038).

### Adverse events

Participants reported twelve adverse events that were at least possibly related to the intervention. None of these were serious and most involved musculoskeletal symptoms. There were no serious adverse events, no adverse events related to coronary artery disease, and no significant differences in adverse events between groups.

## Discussion

Varying the types of goals participants received did not substantially change patterns of walking among sedentary individuals with type 2 diabetes using an automated Internet-mediated walking program. In particular, SG did not result in more bout steps than LG, primarily because most of the successful LG participants chose to reach their total step goals by increasing their bout steps. The bout step increases of almost 2000 steps indicate that participants walked an average of one mile more every day at the end of the program than they were walking at the beginning of the program. At a pace of 3 miles per hour, that would be an additional 20 minutes of moderate intensity activity each day. Because many of the successful participants in our LG group did increase their bouts of moderate intensity walking, presumably they gained the same health benefits that would have resulted from success in a program using structured goals targeting moderate intensity bouts of walking.

It is perhaps not surprising that LG induced increases in bout steps for many participants. With low goals, it may be easy to add in a few extra steps here and there throughout the day, rather than going on a sustained moderate-intensity walk. However, as goals increase, it becomes more and more difficult to achieve success without incorporating sustained moderate-intensity bouts of walking into the day.

Participants who received structured goals were less satisfied with and less adherent to the intervention. This difference was demonstrated in participant responses to closed-end satisfaction questions, in coded themes from answers to open-ended questions, and in the data on adherence to wearing the pedometer. These differences in participant satisfaction are likely to impact negatively long-term adherence to a pedometer-based walking program. While our pilot testing suggested that SG might not be as well received as LG, we were somewhat surprised by the magnitude of the difference in satisfaction between the two groups, particularly considering that many components of the two interventions were identical. Themes identified in response to open-ended questions suggest that the lack of continuous reinforcement for SG participants was part of what caused dissatisfaction.

In another pedometer-based intervention study, Le Masurier (2003) found that about 49% of individuals who reached a 10,000 step target did not meet the minimum criteria of 30 minutes activity in bouts lasting for at least 10 minutes [17]. Because Le Masurier did not report baseline bout activity and because bouts of activity were measured using an accelerometer, the increase in the proportion of steps that occurred during bouts could not be determined. Goals assigned to the Stepping Up to Health participants were based on actual steps taken during the previous week, and were for the most part significantly lower than the 10,000 step goals often used for thinner, fitter, and healthier individuals. In the LG group, most participants who increased their total steps also increased their bout steps even with these relatively low LG.

This pilot study was designed to compare two versions of the Stepping Up to Health intervention, and included no true control group for evaluating the impact of Stepping Up to Health on patients' walking. Although pre-post increases in walking may reflect a Hawthorne effect, the change in step counts was substantial, and supports other evidence that pedometer-based walking programs are effective interventions for increasing physical activity among people with diabetes [17].

Despite the fact that all participants in this pilot study had type 2 diabetes, and thus were at high risk for adverse cardiovascular events, none of the participants experienced any serious or cardiovascular-related adverse events while participating in this unsupervised, unmonitored home-based walking program. This supports previous research demonstrating that moderate intensity physical activity programs are safe even in high-risk groups [30,31].

The results of this pilot study must be interpreted in light of its limitations. The most important limitations of this study are the short duration of the intervention and the small number of participants. Increasing walking for only six weeks may produce transient improvements in some risk factors, but unless it is sustained over a significantly longer time, the benefits are not likely to persist. The number of participants was relatively small yet provided a sufficient sample size to detect statistically significant differences. Nonetheless, it would be reassuring to see these results replicated in a larger sample. Recruitment for this trial was limited to those who had computer and Internet access. Because of this, our results may not be generalizable to those without Internet access or to those with lower socioeconomic status. Additionally, those randomized to the structured goals group were slightly more active at baseline than those randomized to the lifestyle goals group. By calculating individually tailored step count goals based on objectively measured walking history, we minimized the effect of this baseline difference between group comparisons of step count increases. Finally, the definition of a bout step – steps taken during a walk lasting for at least 10 minutes at a pace of at least 60 steps per minute – is a relatively low-intensity threshold, approximately 2 miles per hour, and it might be too low of an intensity threshold to induce a cardio-respiratory training effect on the more fit participants in the trial.

Currently available and affordable information technology has made it possible to automate personally tailored physical activity interventions that can be delivered at low marginal cost over the Internet. However, we know little about how to design these interventions to promote sustained adherence and to optimize health outcomes. There are many more unanswered questions in the design of such complex systems. One critical question is how to help those 57% who were non-responders in this study – those who did not significantly increase their total steps to start walking. Larger and longer studies are needed to provide further evidence-based optimization of automated internet-mediated walking programs.

## Conclusion

Pedometer-based walking programs that emphasize total accumulated step counts are more acceptable to participants and are as effective at increasing moderate intensity bouts of physical activity as programs that use structured goals. Thus, pedometer-based walking programs that target LG (total step) rather than SG (bout step) may result in similar health benefits and in more satisfied participants.

## Competing interests

The author(s) declare that they have no competing interests.

## Authors' contributions

CR supervised all aspects of the study including study design, obtaining funding, intervention development, participant recruitment, data analysis and manuscript preparation. KM and LM participated in participant recruitment, literature searches, data management and manuscript preparation. AJ, study coordinator for Stepping Up to Health, recruited subjects, reported to regulatory bodies, managed data, assisted with data cleaning, and participated in manuscript preparation. LF, a senior coordinator, wrote the protocol for the randomized controlled trial, supervised subject recruitment, ensured regulatory compliance, and participated in manuscript preparation. AS participated in the study design and performed the statistical analysis. VS participated in the study design, intervention development and manuscript preparation. JP participated in the study design, mentoring and manuscript preparation. All authors read and approved the final manuscript.

**Table 5:** Individual participant walking patterns.

	**All Participants**	**Lifestyle Goals Group**	**Structured Goals Group**
**N**	30	17	13
Increased both total and bout steps	37%	35%	38%
Increased only total steps	7%	12%	0%
Increased neither total nor bout steps	57%	53%	62%
	100%	100%	100%
